# Identification of new arylamine N-acetyltransferases and enhancing 2-acetamidophenol production in *Pseudomonas chlororaphis* HT66

**DOI:** 10.1186/s12934-020-01364-7

**Published:** 2020-05-19

**Authors:** Shuqi Guo, Yunxiao Wang, Wei Wang, Hongbo Hu, Xuehong Zhang

**Affiliations:** 1 State Key Laboratory of Microbial Metabolism, School of Life Sciences and Biotechnology, Shanghai Jiao Tong University, Shanghai, 200240 China; 2 National Experimental, Teaching Center for Life Sciences and Biotechnology, Shanghai Jiao Tong University, Shanghai, 200240 China

**Keywords:** *Pseudomonas chlororaphis*, Arylamine N-acetyltransferase, 2-Acetamidophenol, Biosynthesis, Aromatic chemicals

## Abstract

**Background:**

2-Acetamidophenol (AAP) is an aromatic compound with the potential for antifungal, anti-inflammatory, antitumor, anti-platelet, and anti-arthritic activities. Due to the biosynthesis of AAP is not yet fully understood, AAP is mainly produced by chemical synthesis. Currently, metabolic engineering of natural microbial pathway to produce valuable aromatic compound has remarkable advantages and exhibits attractive potential. Thus, it is of paramount importance to develop a dominant strain to produce AAP by elucidating the AAP biosynthesis pathway.

**Result:**

In this study, the active aromatic compound AAP was first purified and identified in gene *phzB* disruption strain HT66Δ*phzB*, which was derived from *Pseudomonas chlororaphis* HT66. The titer of AAP in the strain HT66Δ*phzB* was 236.89 mg/L. Then, the genes involved in AAP biosynthesis were determined. Through the deletion of genes *phzF*, *Nat* and *trpE*, AAP was confirmed to have the same biosynthesis route as phenazine-1-carboxylic (PCA). Moreover, a new arylamine N-acetyltransferases (NATs) was identified and proved to be the key enzyme required for generating AAP by in vitro assay. *P. chlororaphis* P3, a chemical mutagenesis mutant strain of HT66, has been demonstrated to have a robust ability to produce antimicrobial phenazines. Therefore, genetic engineering, precursor addition, and culture optimization strategies were used to enhance AAP production in *P. chlororaphis* P3. The inactivation of *phzB* in P3 increased AAP production by 92.4%. Disrupting the phenazine negative regulatory genes *lon* and *rsmE* and blocking the competitive pathway gene *pykA* in P3 increased AAP production 2.08-fold, which also confirmed that AAP has the same biosynthesis route as PCA. Furthermore, adding 2-amidophenol to the KB medium increased AAP production by 64.6%, which suggested that 2-amidophenol is the precursor of AAP. Finally, by adding 5 mM 2-amidophenol and 2 mM Fe^3+^ to the KB medium, the production of AAP reached 1209.58 mg/L in the engineered strain P3Δ*phzB*Δ*lon*Δ*pykA*Δ*rsmE* using a shaking-flask culture. This is the highest microbial-based AAP production achieved to date.

**Conclusion:**

In conclusion, this study clarified the biosynthesis process of AAP in *Pseudomonas* and provided a promising host for industrial-scale biosynthesis of AAP from renewable resources. 
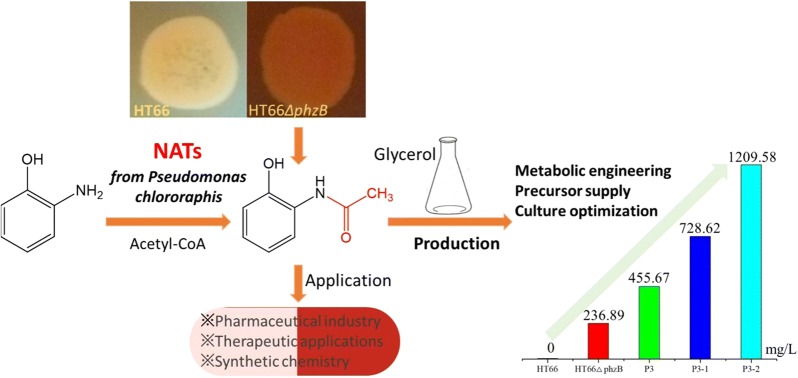

## Background

Aromatic compounds are versatile chemicals used in chemicals, foods, pharmaceuticals, materials, and etc. [1–3]. Currently, most of them are derived from benzene, toluene, xylene and manufactured from petroleum [2, 4, 5]. 2-Acetamidophenol (AAP), also known as N-(2-hydroxyphenyl)-acetamide or O-acetaminophenol, is an aromatic compound derived from salicylic acid [6]. Owing to its potential antifungal, anti-inflammatory, antitumor, anti-platelet, and anti-arthritic activities [7–10], and because it is less toxic than aspirin, AAP has been widely used in the pharmaceutical industry, therapeutic applications and synthetic chemistry [11–13]. Currently, since the biosynthesis of AAP is not yet fully understood, AAP is mainly produced by chemical synthesis [8]. Notably, metabolic engineering of natural microbial pathway to produce valuable aromatic compounds has remarkable advantages and exhibits attractive potential recently [1–5, 14]. Thus, it is of importance to develop a dominant strain to produce AAP by elucidating the AAP biosynthesis pathway.

At present, microbial fermentation provides an alternative way to manufacture chemicals from renewable sources such as biomass feedstock, which is of great significance for sustainable development [2, 4, 14, 15]. Natural AAP production was first reported in the liquid culture of strain *Pseudomonas fluorescens* 2–79, but the maximum titer of AAP was only 50 mg/L [9], that is too low to meet the current demand for industrial production. AAP was subsequently found in various strains, including *P. pyrrocinia* ATCC 15,958 [16], *Fusarium verticillioides* [17], co-culture strains *Actinokineospora* sp. EG49 and *Nocardiopsis* sp. RV163 [18], and *Penicillium* sp. SCSIO 05705 [19]. However, these previous studies did not report the titer of AAP. Therefore, although these reports provide an alternative method to produce AAP from biomass feedstock, there is still an urgent need to develop an efficient host for AAP production.

Currently, there are different hypotheses of the AAP biosynthesis. Lehninger et al. reported that AAP is derived from tyrosine and phenylalanine through the tyrosine biosynthesis pathway [20]. Lübbe et al. reported that AAP is converted from anthranilic acid [16]. Winkler et al. suggested that AAP arises from chorismic acid via amino-deoxyisochorismic acid (ADIC) and 3-hydroxyanthranilic acid (HAA) [21]. Moreover, Slininger et al.’s results suggested AAP and phenazine-1-carboxylic (PCA) likely share the same biosynthesis pathway [9]. Overall, although there are different findings regarding the precursors of AAP, it is commonly believed that AAP is derived from the shikimate pathway. Meanwhile, it has been reported that NATs (N-acetyltransferases) can catalyze the transfer of an acetyl group from acetyl-CoA to the free amino groups of arylamines [22]. Various arylamines such as 2-aminophenol, 3-aminobenzoic acid [23], and *para*-amino salicylic acid [24] have been confirmed to belong to the substrates of NATs. According to the structure of AAP and the related literature [19, 23, 25], NATs should be also involved in the AAP biosynthesis pathway. Many NATs from different strains, including *P. aeruginosa* [25], *Streptomyces griseus* [23], and *Mycobacterium smegmatis* [24], have been reported, but research on the arylamine N-acetyltransferase coming from *P. chlororaphis* is still lacking.

*P. chlororaphis* HT66 is an environmentally friendly, non-pathogenic biocontrol bacterium that produces phenazine-1-carboxamide (PCN) in high titers [26, 27]. Our previous research reported that *P. chlororaphis* HT66 possesses huge potential for producing compounds derived from the shikimate pathway. Additionally, we constructed *P. chlororaphis* HT66 engineered strains that could produce PCN at a titer of 1.80 g/L [26], muconic acid of 3.37 g/L [28], and arbutin of 6.79 g/L [29] from glycerol. Notably, due to its well-characterized physiology and genetics, *P. chlororaphis* has been identified as a desirable bacterium to be developed as a platform strain for the industrial-scale production of compounds derived from the shikimate pathway [27–31]. Therefore, the *P. chlororaphis* HT66 strain may be also a robust host to synthesize AAP by utilizing renewable resources.

In this work, the natural active aromatic compound AAP was first isolated from the engineered strain HT66Δ*phzB*. Subsequently, the genes involved in the AAP biosynthesis pathway were determined by gene deletion and in vitro assays. A new NATs as the key enzyme in AAP biosynthesis was also identified. Furthermore, rational metabolic engineering strategies and a modified medium were used to enhance AAP production in *P. chlororaphis* HT66. The results of this study demonstrated a feasible and efficient platform for the synthesis of AAP from renewable resources.

## Results and discussion

### Inactivation of gene *phzB* in *P. chlororaphis* HT66

**Fig. 1:**
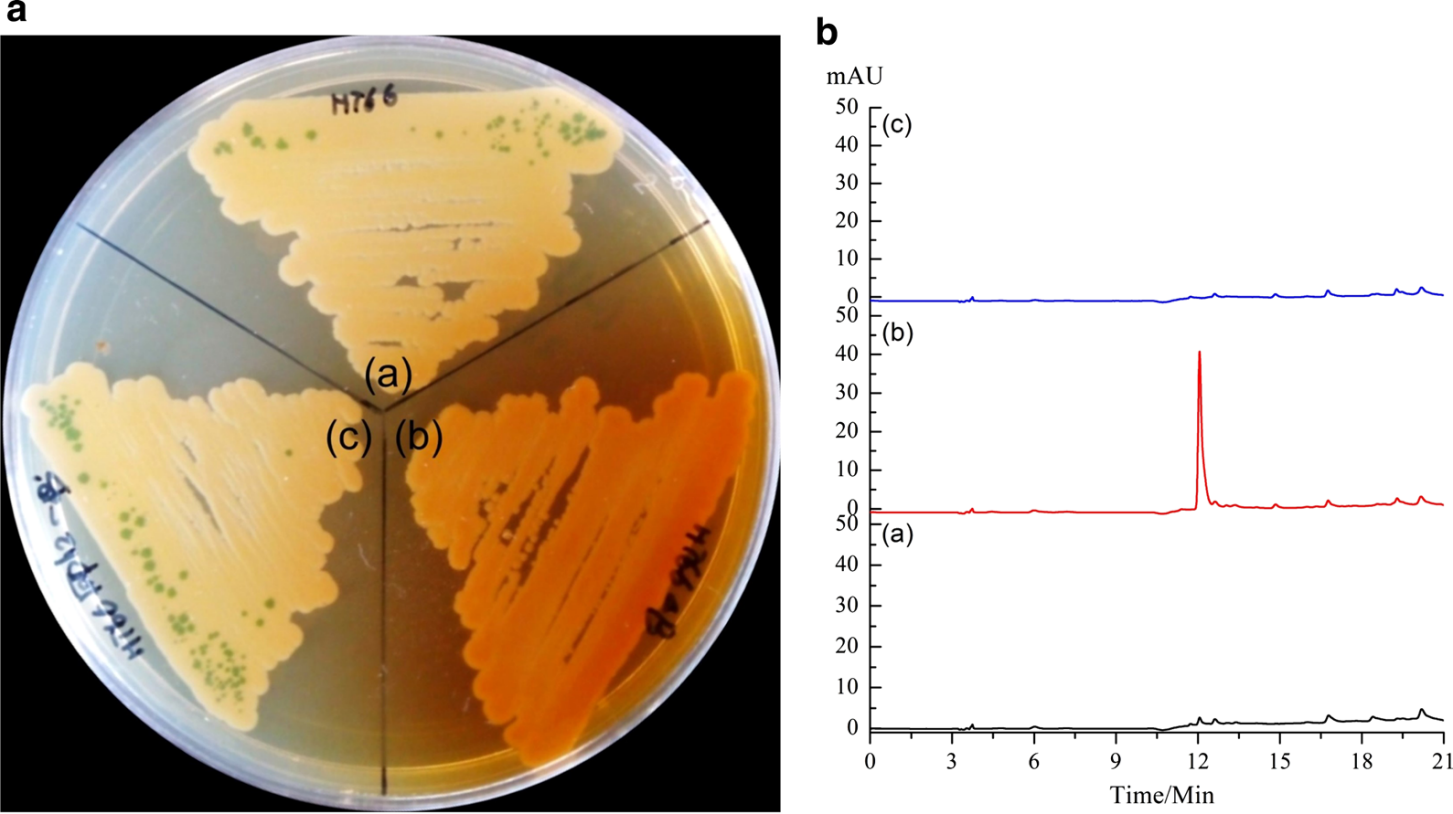
Phenotype and HPLC profile of the *P. chlororaphis* HT66 wild-type and gene *phzB* derived mutants. **A** phenotype: (a) wild-type strain *P. chlororaphi* HT66, (b) *phzB*-inactivated mutant HT66Δ*phzB*, and (c) *phzB* genetic complementation strain HT66Δ*phzB*-pBBR phz’-*phzB*. **B** HPLC profile of wild-type and gene *phzB* derived mutants

Our previous study reported the function of gene *phzA* in the biosynthesis of phenazine-1,6-dicarboxylic acid in *P. chlororaphis* HT66 [32]. Based on this, the gene *phzB* involved in phenazine biosynthesis was deleted to test its function in *P. chlororaphis* HT66. The mutant of the *phzB*-disrupted strain HT66Δ*phzB*, as well as the *phzB* genetic complementation strain HT66Δ*phzB*-pBBR *phz*’-*phzB*, was confirmed by polymerase chain reaction (PCR) and DNA sequencing. When growing all the mutants on solid KB medium, the phenotypes of all strains showed obvious distinctions. The strain HT66 produced the green crystal PCN, whereas the mutant HT66Δ*phzB* produced gray-red colonies (Fig. 1a). Meanwhile, the genetic complementation strain HT66Δ*phzB*-pBBR *phz*’-*phzB* restored the ability to produce PCN. Then, the high-performance liquid chromatographs (HPLC) profiles of HT66 and HT66Δ*phzB* were analyzed. As shown in Fig. 1b, in comparison to wild-type HT66, a new peak was found in the mutant HT66Δ*phzB*. Subsequently, the purification of the new compound was performed.

### Purification and structural elucidation of the new metabolite

**Fig. 2:**
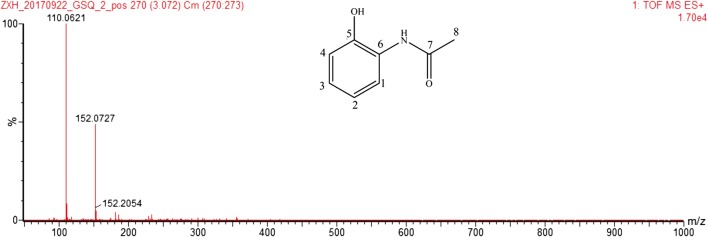
Mass spectrometry of purified compound AAP. The calculated mass of AAP was m/z 152.0706 for [M (C_8_H_9_NO_2_) + H]^+^. The exact mass m/z 152.0727 was purified compound AAP. The Mass m/z 110.0621 was AAP losing the acetyl group*

After purification of the new compound by series steps, a gray-red solid was obtained (Additional file 1: Figure S1). This solid was analyzed by mass spectrometry and nuclear magnetic resonance (NMR). The liquid chromatogram-high resolution mass spectrometry (LC-HRMS) result showed that the exact mass of this compound was *m/z* 152.0727 for [M (C_8_H_9_NO_2_) + H]^+^ (Fig. 2). The chemical structure of the metabolite was also identified by both ^1^H-NMR and ^13^C-NMR. The ^1^H-NMR (600 MHz, dimethyl sulfoxide) spectrum was mainly as follows: δ 7.667 (d, *J *= 7.8 Hz), 6.752 (tt, *J *= 7.8 Hz), 6.931 (tt, *J *= 8.4 Hz), 6.852 (dd, *J *= 8.4 Hz), 2.090 (s), -NH 9.308 (broad), and –OH 9.755 (broad) (Additional file 1: Table S1). The carbon ^13^C NMR (151 MHz, DMSO) spectrum was mainly as follows: δ 122.37 (C1), 118.95 (C2), 124.63 (C3), 115.89 (C4), 147.87 (C5), 126.39 (C6), 168.99 (C7), and 23.60 (C8) (Additional file 1:Table S1). Also, the compound was analyzed by LC/MS/MS and a series of the two-dimensional spectra (Additional file 1: Figures S1–S7). In conclusion, the structure of this compound was confirmed to be 2-acetamidophenol. Although natural 2-acetamidophenol has been reported in the strains *P. pyrrocinia* ATCC 15958 [16], *P. fluorescens* 2-79 [9], *Fusarium verticillioides* [17], co-culture strains *Actinokineospora* sp. EG49 and *Nocardiopsis* sp. RV163 [18], and *Penicillium* sp. SCSIO 05705 [19], this is the first time natural 2-acetamidophenol has been discovered in the genetically engineered strain.

### Identification of genes involved in AAP biosynthesis

**Fig. 3:**
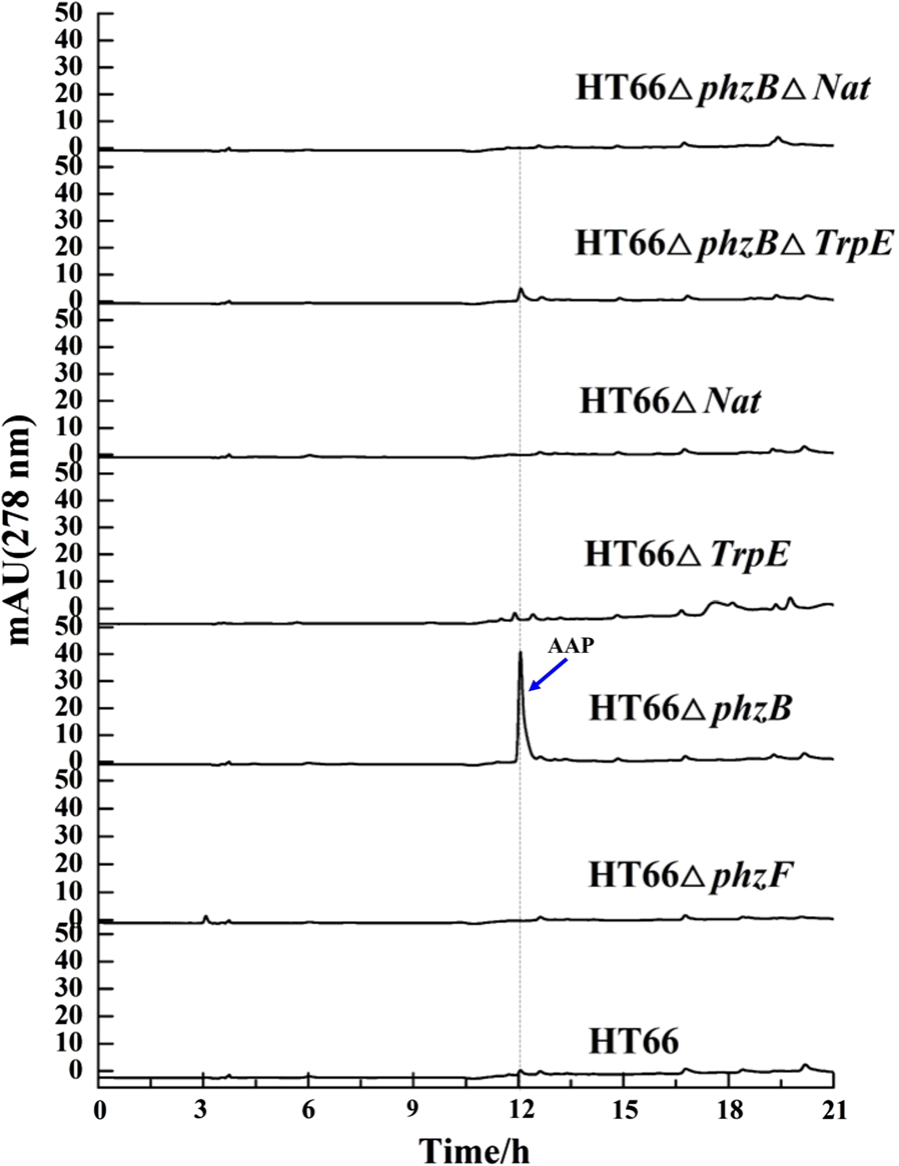
The genes involved in AAP biosynthesis pathway in *P. chlororaphis* HT66 analyzed by gene deletion

Based on the gene deletion results and the available literature, AAP biosynthesis was suggested to be involved in the phenazine-1-carboxylic acid (PCA) biosynthesis pathway [9]. Also, the results proved that five conserved enzymes (PhzE, PhzD, PhzF, PhzB, and PhzG) in all phenazine-producing operons sequentially convert chorismic acid to PCA [33]. Since AAP was found to be secreted by HT66Δ*phzB*, we first assumed that the phenazine biosynthesis pathway was also responsible for AAP biosynthesis. To confirm the encoded genes involved in the biosynthesis of AAP, we first deleted gene *phzF* in *P. chlororaphis* HT66 (Fig. 3). However, the strains HT66Δ*phzF* and HT66Δ*phzB*Δ*phzF* were both AAP-deficient and just accumulated *trans*-2,3-dihydro-3-hydroxyanthranilic acid (DHHA) (Fig. 3, Additional file 1: Table S2). A previous study of phenazine biosynthesis has made it clear that DHHA can be converted to 6-amino-5-oxocyclohex-2-ene-1-carboxylic acid (AOCHC) by *phzB* [34]. In addition, in strain HT66Δ*phzA* and HT66Δ*phzB*, the production of AAP increased with the decrease of phenazine compounds production [32] (Additional files 1: Figure S8, Table S2). Combining our results with Slininger et al.’s report [9], it was revealed that AAP and PCA shared the same pathway during biosynthesis. In the wild-type strain *P. chlororaphis* HT66, the genes *phzA*, *phzB* and *phzG* together accelerated the accumulation of PCA and made the production of AAP show a positive correlation with PCA [9, 32]. But in *phzA*, *phzB* deletion strains, HT66Δ*phzA*, HT66Δ*phzB* and HT66Δ*phzA*Δ*phzB*, the intermediate was mainly converted to AAP, and the PCA production dropped obviously (Additional file 1: Figure S8). This may also reveal that the robust ability of PCN production caused all the intermediates to be converted to PCN, so AAP was undetectable in the wildtype [26].

**Fig. 4:**
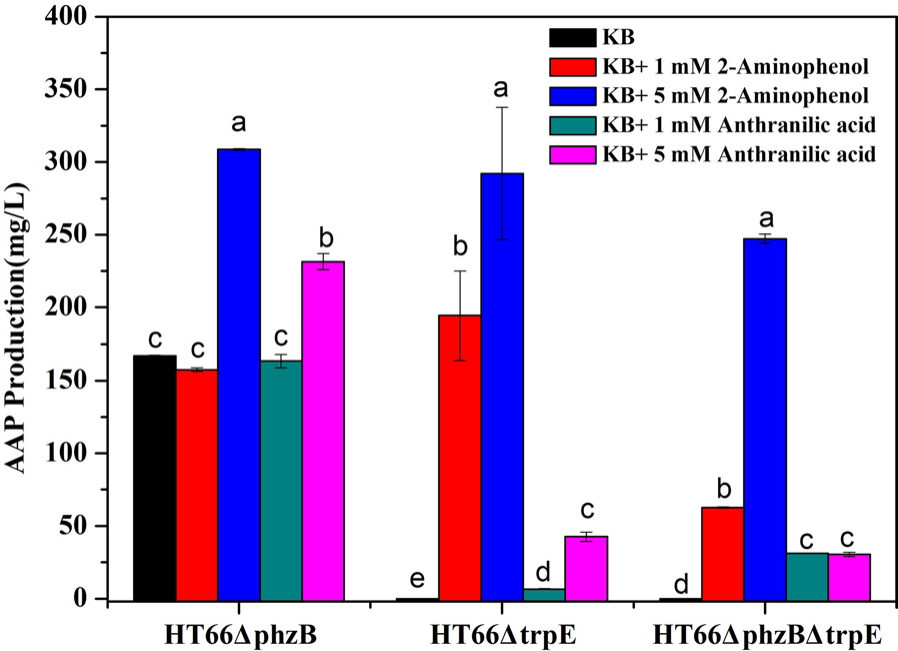
The production of AAP under the condition of adding different concentrations of 2-aminophenol and Anthranilic acid

Moreover, Lehninger reported that AAP can be derived from the tryptophan biosynthesis pathway. Thus, we constructed the mutant HT66Δ*trpE*. As shown in Fig. 3, when gene *trpE* was inactive, the strain HT66Δ*trpE* was also AAP-deficient. We also got the double gene deletion strain HT66Δ*phzB*Δ*trpE*; in comparison with strain HT66Δ*phzB*, the production of AAP in HT66Δ*phzB*Δ*trpE* was significantly lower and barely detectable (Fig. 3, Additional file 1: Table S2). These results may suggest that gene *trpE* is not essential but can substantially enhance the biosynthesis of AAP. Meanwhile, the structure of AAP revealed that NATs should be involved in the AAP biosynthesis [19, 23, 25]. Next, through protein sequence alignment (Additional file 1: Figure S9), we found a candidate enzyme NATs (coded by gene *Nat*, accession number NZ_ATBG01000008) in the *P. chlororaphis* HT66 genome (Additional files 1: Table S3, Figure S9). When we deleted gene *Nat*, as predicted, the strains HT66Δ*Nat* and HT66Δ*phzB*Δ*Nat* both lost the ability to produce AAP (Fig. 3). Our results may also predict that AAP is generated from 2-aminophenols by the acetylation of NATs, which is similar with the function of arylamine N-acetyltransferase in *Streptomyces griseus* [23]. In addition, it is worth mentioning that Lübbe et al. [16] suggested that anthranilic acid may be one intermediate to generate AAP. In consideration of the similar structure of anthranilic acid and 2-aminophenol, we separately added each of these two compounds to the medium to test the AAP production. Notably, when 2-aminophenol was added to the KB medium, the AAP production improved obviously (Fig. 4). Also, under the catalysis of NATs, the strains HT66Δ*trpE* and HT66Δ*phzB*Δ*trpE* both accumulated AAP (Fig. 4). These results not only suggested that 2-aminophenol may be the main intermediate in AAP biosynthesis but also revealed that the key function of NATs is converting 2-aminophenol to AAP in *P. chlororaphis* HT66. Interestingly, when anthranilic acid was added to the KB medium, the AAP production also increased slightly in strains HT66Δ*phzB* and HT66Δ*TrpE*. It may be that some anthranilic acid was converted to 2-aminophenol in *P. chlororaphis* HT66 or the anthranilic acid was conducive to increasing the concentration of the essential amino acid tryptophan in the medium. In addition, there is a possible regulatory effect that anthranilic acid shows the feedback inhibition of the enzyme in AAP biosynthesis pathway, due to the similarity structure with 2-aminophenol.

### Purification and in vitro assay of NATs

**Fig. 5:**
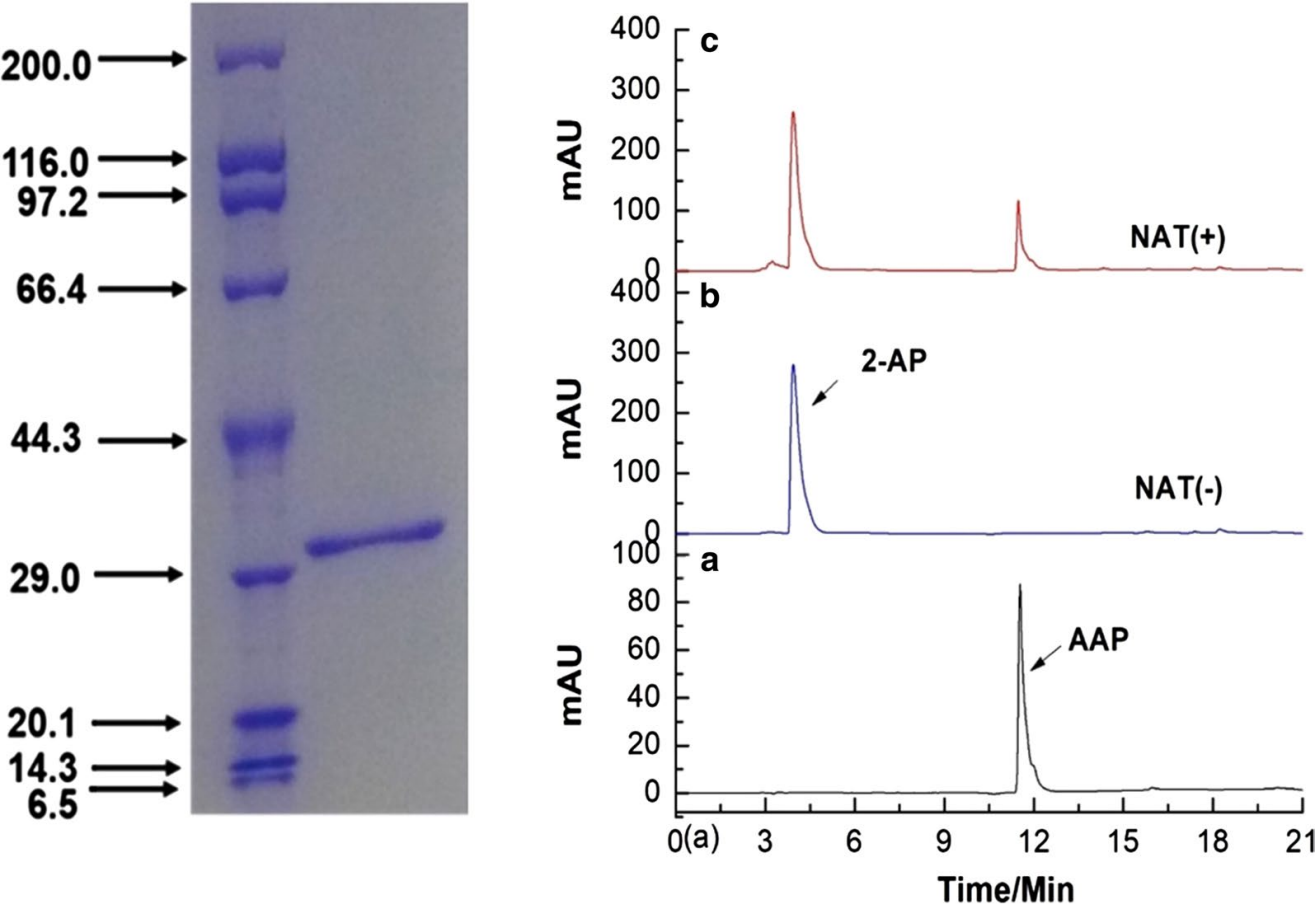
In vitro catalysis of NATs to 2-aminophenol. **A** SDS-PAGE of protein NATs, 32.2KDa. **B** (a) The standard of AAP, (b) catalysis system without NATs, and (c) catalysis system with NATs. AAP: 2-acetamidophenol; 2-AP: 2-aminophenol

**Fig. 6:**
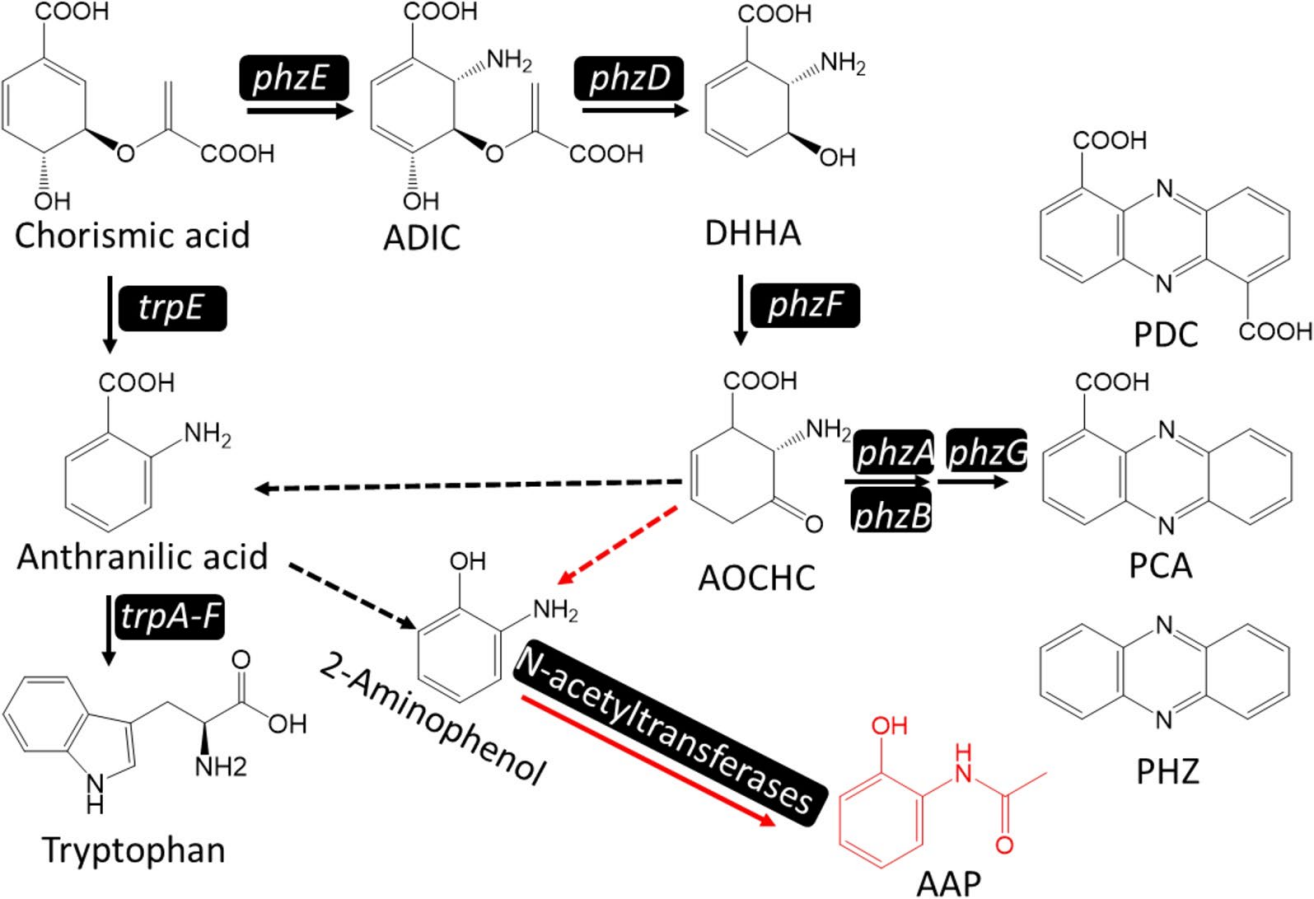
Proposed biosynthesis pathway of AAP in *P. chlororaphis* HT66. ADIC: amino-deoxyisochorismic acid; DHHA: *trans*-2,3-dihydro-3-hydroxyanthranilic acid; AOCHC: 6-amino-5-oxocyclohex-2-ene-1-carboxylic acid; PDC: phenazine-1,6-dicarboxylic acid; PCA: phenazine-1-carboxylic; PHZ: phenazine; AAP: 2-acetamidophenol

To confirm that NATs from *P. chlororaphis* HT66 can catalyze 2-aminophenol, we expressed the gene *Nat* in *E. coli* BL21 and purified the encoded protein NATs (Fig. 5a). Notably, the in vitro catalysis result showed that NATs can convert 2-aminophenol to AAP (Fig. 5b). Moreover, we also purified protein PhzF and TrpE (Additional file 1: Figure S10). Unfortunately, the enzyme mixture of PhzF, TrpE and NATs could not convert DHHA to AAP in vitro. These results suggested that in addition to PhzF, TrpE and NATs, there may be other genes involved in converting DHHA to AAP (Additional file 1: Table S4). Although our results cannot fully elucidate the biosynthesis process of AAP in *P. chlororaphis* HT66, this study clearly indicates that AAP and PCA share the same biosynthesis pathway (Fig. 6). The presence of NATs in *P. chlororaphis* HT66 was key to the generation of AAP. This is the first report of NATs in *P. chlororaphis*. The results of the experiment in which 2-aminophenol was added to the KB medium revealed that NATs has high catalysis activity and possesses the potential for industrial catalysis application.

### Production of 2-Acetamidophenol in *P. chlororaphis* HT66

AAP is extremely versatile, not only showing strong antifungal activity in agricultural applications [9] but also exhibiting promising characteristics for medical applications, such as anti-proliferative, anti-platelet, anti-inflammatory, and antiarthritic activities [6, 8, 35]. Moreover, the above studies show that AAP and phenazine share the same biosynthesis pathways, and in the past, we have conducted many studies on improving the titer of phenazines in *P. chlororaphis* HT66 [26, 28, 30]. Based on these studies, we improved the production of AAP in *P. chlororaphis* HT66 by genetic engineering, precursor addition, and medium optimization. In the wild-type *P. chlororaphis* HT66, AAP was not detected in the KB medium at 28 °C for 72 h (Table 1, entry 1). After the deletion of gene *phzB* in *P. chlororaphis* HT66, AAP was isolated and identified. After 72 h culture, the mutant strain produced AAP at a concentration of 236.89 mg/L (Table 1, entry 2). As the AAP biosynthesis pathway was in connection with the phenazine biosynthesis pathway, the mutagenesis mutant P3 was a high PCA derivative (PCN) producing strain. We also deleted gene *phzB* in P3. Notably, in comparison with HT66Δ*phzB*, the concentration of AAP increased by 92.4% and reached 455.67 mg/L in strain P3Δ*phzB* (Table 1, entry 3).

**Table 1:** Production of 2-acetamidophenol in the *P. chlororaphis* HT66 wild-type and derived strains (*P *< 0.05)

Entry	HT66	Fe^3+^ (mM)	2-aminophenol (mM)	AAP concentration (mg/L)
1	WT			0.00 ± 0.00 g
2	HT66Δ*phzB*			236.89 ± 4.28 f
3	P3Δ*phzB*			455.67 ± 59.59 e
4	P3Δ*phzB*	2		750.13 ± 0.97 c
5	P3Δ*phzB*Δ*lon*Δ*pykA*Δ*rsmE*			728.62 ± 13.99 d
6	P3Δ*phzB*Δ*lon*Δ*pykA*Δ*rsmE*	2	1	1130.86 ± 22.42 b
7	P3Δ*phzB*Δ*lon*Δ*pykA*Δ*rsmE*	2	5	1209.58 ± 5.74 a

It has been reported that ferric iron (Fe^3+^) has a positive effect on the production of PCA [36]. One of our previous studies found that the PCN production of P3 increased by 54.2% when the KB media was amended with 2.0 mM FeCl_3_ [27]. When the same media was used to culture P3Δ*phzB*, the concentration of AAP increased by 64.6% and up to 750.13 mg/L (Table 1, entry 4). This result may also confirm that the AAP biosynthesis pathway is the same as the PCA biosynthesis route. To further enhance the AAP production, metabolic engineering strategies were employed. In *P. chlororaphis* P3, the disruption of negative regulatory genes and the blocking of the competitive pathway are very effective ways to enhance the production of PCA and 2-hydroxyphenazine [26, 28, 31, 36, 37]. Moreover, ATP-dependent protease Lon and translational repressors RsmE are involved in the negative regulation of phenazines in *P. chlororaphis* [31]. Also, pyruvate kinase *pykA* is one of the main enzymes involved in consuming the key metabolic precursor phosphoenolpyruvate (PEP) of PCA biosynthesis, so blocking this PEP competitive pathway can enhance 2-hydroxyphenazine availability [31, 38]. On this basis, we deleted genes *lon*, *rsmE*, and *pykA* sequentially and got the strain P3Δ*phzB*Δ*lon*Δ*pykA*Δ*rsmE*. After 72 h incubation, the concentration of AAP increased by 59.9% and reached 728.62 mg/L (Table 1, entry 5).

The addition of precursor is a simple and efficient strategy to improve the secondary metabolite production in fermentation [39, 40]. Arbutin production was increased 54-fold in *P. chlororaphis* P3-Ar5 strain, and up to the final title of 6.79 g/L, by adding the precursor 4-hydroxybenzoic acid [28]. The production of compound Alteramide B was increased 89.4%, and the highest concentration 893.32 mg/L was obtained by adding 5 g/L arginine precursor in the strain *Lysobacter enzymogenes* OH11 [39]. When 2 mM FeCl_3_ and  1 mM 2-Amniophenol were added to the KB culture medium, 1130.86 mg/L AAP was detected (Table 1, entry 6). In addition, high concentrations of the substrate can generate feedback inhibition of synthetase [41]. Finally, 1209.58 mg/L AAP was detected when 2 mM FeCl_3_ and 5 mM 2-amniophenol were added to the KB medium (Table 1, entry 7).

In a previous report, although AAP was first isolated from *P. fluorescens* 2–79, the production was only 50 mg/L [9]. In comparison with *P. fluorescens* 2–79, AAP production was increased 24-fold in our engineered strain P3Δ*phzB*Δ*lon*Δ*pykA*Δ*rsmE*. Moreover, 2-aminophenol is quite inexpensive and is commercially available, so it can be used as a raw material for large-scale production of AAP.

## Conclusions

In this study, aromatic compound 2-acetamidophenol was firstly isolated and identified in *P. chlororaphis* HT66Δ*phzB*. A new NATs converting 2-amidophenol to AAP was confirmed. Through gene deletion and enzyme catalysis, AAP was confirmed to share a biosynthesis pathway with PCA. Then, strategies including metabolic engineering, precursor addition, and culture optimization were used to enhance AAP production in the high phenazine producing strain *P. chlororaphis* P3. Finally, AAP production was substantially improved to 1209.58 mg/L in the genetically engineered strain P3Δ*phzB*Δ*lon*Δ*pykA*Δ*rsmE* by adding 5 mM 2-amidophenol and 2 mM Fe^3+^ to the KB medium. This is the highest bio-production of AAP achieved to date. This study thus elucidates the biosynthesis of AAP in *P. chlororaphis* and provides a possible green method to produce this valuable aromatic compound.

## Materials and methods

### Bacterial strains

*P. chlororaphis* HT66 (CCTCC, M2013467) was cultured in the KB medium at 28 °C. *P. chlororaphis* P3 was obtained by subjecting strain HT66 to multiple rounds of chemical mutagenesis and selection [26, 27]. *Escherichia coli* DH5α and S17 were cultivated in LB medium at 37 °C. If necessary, kanamycin and ampicillin were used in the medium (Table 2).

**Table 2:** Strains and plasmids used in this study

Strains and plasmids	Relevant gene type	Reference/source
Strains		
DH5α	*E. coli* F^−^Ф80*lacZ*ΔM15Δ(*lacZYA*-*argF*) U169 *recA1 endA1 hsdR17* (rk^−^ mk^−^) *phoA supE44 thi*^−*1*^*gyrA96* relA1	Lab stock
*E. coli* S17-1 (λpir)	Res^−^ pro mod^+^ integrated copy of RP4, mob^+^, used for incorporating constructs into *P. chlororaphis*	Lab stock
BL21(DE3)	Host strain for pET28a	Invitrogen
HT66	*P. chlororaphis* HT66 wild-type, Amp^r^ Sp^r^	This study
P3	A mutant from HT66 with a high PCN production, Amp^r^ Sp^r^	This study
HT66Δ*phzB*	*phzB* in-frame deletion mutant of HT66	This study
HT66Δ*phzF*	*phzF* in-frame deletion mutant of HT66	This study
HT66Δ*trpE*	*trpE* in-frame deletion mutant of HT66	This study
HT66Δ*Nat*	*Nat* in-frame deletion mutant of HT66	This study
HT66Δ*phzB*Δ*trpE*	*phzB*, *trpE* double in-frame deletion mutant of HT66	This study
HT66Δ*phzB*Δ*Nat*	*phzB*, *Nat* double in-frame deletion mutant of HT66	This study
HT66Δ*phzB*-pBBRphz’-*phzB*	*phzB* complentation in HT66Δ*phzB*	This study
P3Δ*phzB*	*phzB* in-frame deletion mutant of P3	This study
P3Δ*phzB*Δ*lon*Δ*pykA*Δ*rsmE*	*phzB*, *lon*, *pykA*, *rsmE* in-frame deletion mutant of P3	This study
Plasmid		
pK18mobsacB	Broad-host-range gene replacement vector, *sacB*, Kan^r^	Lab stock
pBBR1MCS	T7 expression vector, Kan^r^	Lab stock
PET-28a (+)	T7 promoter, Kan^r^, expression vector	Novagen
pBBR-phz’-*phzB*	Gene complementation vector, pBBR1MCS containing a 534 bp *phz* promoter fragment and the *phzB* gene cluster, Kan^r^	This study
PET28a (+)-*Nat*	pET28a (+) containing *Nat*	This study

^a^Amp^r^, Sp^r^, and Kan^r^ represent ampicillin, spectinomycin, and kanamycin resistance, respectively

### Construction of non-scar deletion and genetic complementation mutant strains

The gene *phzB* involved in PCN biosynthesis was disrupted using a non-scar deletion method in *P. chlororaphis* HT66 [42]. The flanking regions of gene *phzB* were amplified by PrimerSTAR polymerase (TAKARA). The plasmid pK18mobsacB and strain *E. coli* S17-1 were used [27]. The correct mutant strains were verified by PCR and DNA sequencing. The genes *phzF*, *TrpE*, *Nat*, *lon*, *pykA* and *rsmE* were deleted by the same methods. The *phzB* genetic complementation strain was constructed using the plasmid pBBR1MCS following Jin’s method [27]. The correct gene complementation plasmid was transformed into *P. chlororaphis* by electroporation. Primers used are shown in Additional file 1: Table S5.

### Fermentation of *P. chlororaphis* and its derived mutant strains

*P. chlororaphis* HT66 and its derived strains were cultured, and subsequent fermentation processes were all carried out following our previously established method [31, 32]. To enhance the AAP production, anthranilic acid (synthesized in laboratory) and 2-aminophenol (Energy Chemical, Shanghai, China) were added to the medium. FeCl_3_ (Yonghua Chemical Co., Ltd. Shanghai, China) was also used in the optimization of culture conditions.

### Isolation and identification of AAP

To isolate the new compound, HT66Δ*phzB* was cultivated in the KB medium for 72 h. Then, 1 L of culture broth was collected and extracted three times using ethyl acetate. The organic phase was collected and evaporated to dryness. The dry crude was dissolved in methanol, then separated and purified by reversed phase HPLC with semi-prep C18 column under 278 nm (250 × 10.0 mm, 10 μm; Dikma Co., Ltd., Shanghai, China). The mobile phase was 15% methanol in H_2_O from 0 to 6 min, 50% methanol in H_2_O from 6 to 7 min, 50–90% methanol in H_2_O from 7 to 17 min, 90–15% methanol in H_2_O from 17 to 18 min, and 15% methanol from 18 to 21 min. The flow rate was 3 mL/min [43]. The pure AAP was identified by liquid chromatogram-high resolution mass spectrometry (LC-HRMS) on a Waters ACQUITY LC system (Waters Co., Ltd., Milford, USA). ^1^H and ^13^CNMR spectra were obtained with a Bruker Avance III 600 MHz spectrometer (Karlsruhe, Germany). The solvent was dimethyl sulfoxide (DMSO).

### Quantitative assay for AAP and phenazine production

First, 400 μL of the fermentation supernatant was acidified and extracted with 3.6 mL ethyl acetate. Then the 400 μL organic layer was evaporated, 1 mL of chromatographic grade methanol was used to dissolve the samples, and HPLC was used to analyze [31]. For the quantitative assay of AAP production, an Agilent Eclipse XDB-C_18_ reverse-phase column was used. The mobile phase was 15% methanol in H_2_O from 0 to 6 min, 50% methanol in H_2_O from 6 to 7 min, 50–90% methanol in H_2_O from 7 to 17 min, 90–15% methanol in H_2_O from 17 to 18 min, and 15% methanol from 18 to 21 min. Samples were detected at 278 nm with a flow rate of 1 mL/min. The commercial compound 2-acetamidophenol was used as the standard (Energy Chemical, Shanghai, China). The phenazine production was assayed following our previous method [31].

### Expression of Nat in *E. coli*

The gene *Nat* was amplified from the *P. chlororaphis* HT66 strain’s genomic DNA. The PCR products were ligated into vector pET28a (+) (Transgene Biotech Inc., Beijing, China). After being verified by gene sequencing, the vector was transformed into *E. coli* strain BL21 (DE3) (Transgene Biotech Inc., Beijing, China). *E. coli* BL21 (DE3) harboring the pET28a (+)-*Nat* plasmid was grown at 37 °C to an optical density of 0.4 at 600 nm in LB medium, and then 0.1 mM isopropyl *β*-d-1-thiogalactopyranoside (IPTG) was added. The cells were harvested for protein purification after 16 h of incubation at 16 °C and then purified by Ni–NTA agarose column (Sangon Biotech Inc., Shanghai, China). PhzF and TrpE were expressed and purified in a similar manner.

### In vitro enzyme assays

The enzyme catalysis was determined in a 100 μL mixture consisting of 50 mM TRIS–HCl (pH 7.5), 4 mM 2-aminophenol and 0.4 mM acetyl-CoA (Merck, Germany). The assay was initiated by the addition amount of 10 μg purified NATs to the mixture [25, 26]. The reaction was performed at 28 °C for 30 min and then terminated by the addition of 10 μL of cooled 6 M HCl. The products were detected by HPLC. The same reaction volume was used for the catalysis of DHHA by PhzF, TrpE and NATs together. DHHA was prepared by the method described in our previous study [44], and the concentration of DHHA was 20 mM.

### Statistical analysis

The data were analyzed using SPSS (Statistical Package, Version 18.0). The variables were analyzed using Student’s *t* test. All the results were presented as the mean ± standard deviation (triplicate independent experiments). Origin 8.0 software (Northampton, MA, USA) was used to make the figures. The protein sequence alignment was performed with DNAMAN software (version 6.0).

## Supplementary information

**Additional file 1: Table S1.** Chemical shift summarized from 1H (DMSO) and 13C (DMSO) analyses recorded by 600 MHz NMR spectrometry. **Table S2.** The AAP production in the Pseudomonas chlororaphis HT66 derived strains. **Table S3.** The gene sequence of NATs in Pseudomonas chlororaphis HT66. **Table S4.** In vitro catalysis from DHHA. **Table S5.** Primers used in this study. **Figure S1.** The MS/MS spectrum and the physical form of the purified compound. (A): The MS/MS of standard (up) and the purified compound (down). (B): The physical form of the purified compound. **Figure S2.** The 1H NMR spectra of 2-acetamidophenol (DMSO, 600 MHz). **Figure S3.** The 13C spectrum of spectra of 2-acetamidophenol (DMSO, 151 MHz). **Figure S4.** The HMBC spectrum of 2-acetamidophenol (DMSO, 151 MHz). **Figure S5.** The HSQC spectrum of 2-acetamidophenol (DMSO, 151 MHz). **Figure S6.** The NOESY spectrum of 2-acetamidophenol (DMSO, 400 MHz). **Figure S7.** The COSY spectrum of 2-acetamidophenol (DMSO, 600 MHz). **Figure S8.** Linear correlation of AAP and PCA in HT66 derived strains. **Figure S9.** The protein sequence alignment of arylamine N-acetyltransferase from different strains. **Figure S10.** The protein purification of PhzF and TrpE in Pseudomonas chlororaphis HT66.

## Data Availability

All data generated or analyzed during this study are included in this published article and its additional files.
