# Supplementary material for: Identification of new arylamine N-acetyltransferases and enhancing 2-acetamidophenol production in Pseudomonas chlororaphis HT66
Source: Microb Cell Fact. 2020 May 19;19:105. doi: 10.1186/s12934-020-01364-7 (PMC7236291; doi:10.1186/s12934-020-01364-7)
Supplement: Supplementary file 1 — Additional file 1: Table S1. Chemical shift summarized from 1H (DMSO) and 13C (DMSO) analyses recorded by 600 MHz NMR spectrometry. Table S2. The AAP production in the Pseudomonas chlororaphis HT66 derived strains. Table S3. The gene sequence of NATs in Pseudomonas chlororaphis HT66. Table S4. In vitro catalysis from DHHA. Table S5. Primers used in this study. Figure S1. The MS/MS spectrum and the physical form of the purified compound. (A): The MS/MS of standard (up) and the purified compound (down). (B): The physical form of the purified compound. Figure S2. The 1H NMR spectra of 2-acetamidophenol (DMSO, 600 MHz). Figure S3. The 13C spectrum of spectra of 2-acetamidophenol (DMSO, 151 MHz). Figure S4. The HMBC spectrum of 2-acetamidophenol (DMSO, 151 MHz). Figure S5. The HSQC spectrum of 2-acetamidophenol (DMSO, 151 MHz). Figure S6. The NOESY spectrum of 2-acetamidophenol (DMSO, 400 MHz). Figure S7. The COSY spectrum of 2-acetamidophenol (DMSO, 600 MHz). Figure S8. Linear correlation of AAP and PCA in HT66 derived strains. Figure S9. The protein sequence alignment of arylamine N-acetyltransferase from different strains. Figure S10. The protein purification of PhzF and TrpE in Pseudomonas chlororaphis HT66. [file 12934_2020_1364_MOESM1_ESM.docx]

**Supplemental materials**

Identification of new arylamine N-Acetyltransferases and enhancing

2-acetamidophenol production in *Pseudomonas chlororaphis* HT66

Shuqi Guo^1^, Yunxiao Wang^1^, Wei Wang^1^, Hongbo Hu^1,2^, Xuehong Zhang^1*^

^1^State Key Laboratory of Microbial Metabolism, School of Life Sciences and Biotechnology, Shanghai Jiao Tong University

^2^National Experimental Teaching Center for Life Sciences and Biotechnology, Shanghai Jiao Tong University, Shanghai 200240, China

^*^Corresponding author: Tel.: +86-21-3420-6715; Fax: +86-21-3420-5081

E-mail: xuehzhang@sjtu.edu.cn

**Table Captions**

**Table S1** Chemical shift summarized from ^1^H (DMSO) and ^13^C (DMSO) analyses recorded by 600 MHz NMR spectrometry

**Table S2** The AAP production in the *Pseudomonas chlororaphis* HT66 derived strains

**Table S3** The gene sequence of NATs in *Pseudomonas chlororaphis* HT66

**Table S4** *In vitro* catalysis from DHHA

**Table S5** Primers used in this study

**Figure Captions**

**Figure S1** The MS/MS spectrum and the physical form of the purified compound. (A): The MS/MS of standard (up) and the purified compound (down). (B): The physical form of the purified compound

**Figure S2** The ^1^H NMR spectra of 2-acetamidophenol (DMSO, 600 MHz)

**Figure S3** The ^13^C spectrum of spectra of 2-acetamidophenol (DMSO, 151 MHz)

**Figure S4** The HMBC spectrum of 2-acetamidophenol (DMSO, 151 MHz)

**Figure S5** The HSQC spectrum of 2-acetamidophenol (DMSO, 151 MHz)

**Figure S6** The NOESY spectrum of 2-acetamidophenol (DMSO, 400 MHz)

**Figure S7** The COSY spectrum of 2-acetamidophenol (DMSO, 600 MHz)

**Figure S8** Linear correlation of AAP and PCA in HT66 derived strains

**Figure S9** The protein sequence alignment of arylamine N-acetyltransferase from different strains

**Figure S10** The protein purification of PhzF and TrpE in *Pseudomonas chlororaphis* HT66

**Table S1** Chemical shift summarized from ^1^H (DMSO) and ^13^C (DMSO) analyses recorded by 600 MHz NMR spectrometry

| Position | *δ*_H_ (*J* in Hz) | *δ*_C_ | HMBC (H to C) | COSY (H to H) |
| --- | --- | --- | --- | --- |
| 1 | 7.667, d (7.8) | 122.37 | 3,-NH | 2 |
| 2 | 6.752, tt (7.8/1.2) | 118.95 | 4 | 1, 3 |
| 3 | 6.931, tt (8.4/1.2) | 124.63 | 1 | 2, 4 |
| 4 | 6.852, dd (8.4/1.2) | 115.89 | 2 | 3 |
| 5 | - | 147.87 | 1, 3, -NH | - |
| 6 | - | 126.39 | 2, 4 | - |
| 7 | - | 168.99 | -NH, 8 | - |
| 8 | 2.090, s | 23.60 | - | - |
| -NH | 9.308, b | - | - | - |
| -OH | 9.755, b | - | - | - |

**Table S2** The AAP production in the *Pseudomonas chlororaphis* HT66 derived strains

| Mutant | AAP |
| --- | --- |
| HT66 | - |
| HT66Δ*phzF* | - |
| HT66Δ*phzA* | + |
| HT66Δ*phzB* | + |
| HT66Δ*trpE* | - |
| HT66Δ*Nat* | - |
| HT66Δ*phzB*Δ*trpE* | * |
| HT66Δ*phzB*Δ*Nat* | - |

*The “+” indicates that AAP was detected. The “-” indicate that AAP was not detected. The “*” indicates that AAP production was very low.

**Table S3** The gene sequence of NATs in *Pseudomonas chlororaphis* HT66

| Gene *Nat* in *Pseudomonas chlororaphis* HT66 (accession number NZ_ATBG01000008 ) |
| --- |
| ATGAGTCAGCCCCACCTGATGAACTTCGCGCTTTACCTGCAACGGCTCGGCTACGACAGCGCGCCGCCGCCGACCCTGGCGACCCTGCGCGAGCTGCAGTTGCGCCATACCAGCACCTTCGCCTTCGAAAGCCTGTCGACCCTGTTGCGCGTGCCGGTGCCGATCGATCTGGCCTCGGTGGAACACAAGGTCCTGCATGAGGGCCGTGGCGGCTATTGCTACGAACTCAACCAGATGTACCAGGTGCTGTTGCAGCACCTAGGCTACGAGGCGCGCGGCATTACCGGGCGGGTGGTGATGGGCGGGCCGGAAGATGCCTGGTCCGCGCGCGCTCACCGCCTGGTGCTGCTGACCCTGGATGGCGTGCGTTATGTCTCGGATGTCGGCTTTGGCGGCATGGTGCCCACCGCGCCGTTGCTGCTCGATACCGAGGACGAGCAGGCCACGCCCCACGAGCCCTATCGCATCACCGAGCGTGATGGCAGTTACACCCTGCGCGCCAAGGTCGCCGGCGAATGGCGGGCGATGTATGTATTCGACCTGCAGCGGCAGGAAGCCGTGGATTACGAAATGGGTAACTGGTTCGTATCGACCCACAAGAACTCGCCGTTTCTCGGCCAGTTGAAGGTCGCGCTGACGGGCGCAGGCCTGCGCCGGACCCTTCACAACGGCAGCTACGCCGTGCATCGCCTGGGCCAGGACAGCGAGCGGCGGCAGATCACCGACCCCGACGAACTGATTGCCCTGTTGCAGAACGAGTTCGCCATTCGGGTTCCGCGGCATCCGCGGCTGCGCGAAGTCTTCGGCGAATTGCTCGCGCTGCCGCAACCGGCCTGA |

**Table S4** *In vitro* catalysis from DHHA

| Catalysis | AAP |
| --- | --- |
| DHHA+PhzF | - |
| DHHA+PhzF+TrpE | - |
| DHHA+PhzF+TrpE+Nat | - |
| DHHA+TrpE | - |
| DHHA+PhzF+Nat | - |

* The “-” indicate that AAP was not detected.

**Table S5** Primers used in this study

| primes | Sequence 5’ **→** 3’ | application |
| --- | --- | --- |
| *phzB*-F1 | ccggggatcctctagaTTTACTTTCAAAAAATACCAGCCCA | *phzB* gene deletion |
| *phzB*-R1 | AAGTTCAGTGGTGTCGCTAGCG |  |
| *phzB*-F2 | *AGCGACACCACTGAACTT*GCGCGCCCTGGGTATTCC |  |
| *phzB*-R2 | ggccagtgccaagcttGTGAACCTATGAGTTGCTCCTGGC |  |
| *phzB*-in-F | CCTAATAGCGCAGCGCAACA |  |
| *phzB*-in-R | GGATGCCTTCGCGTTTGATT |  |
| C*phzB*-F1 | cggtatcgataagcttAAGACCCTCAATGTGACAGCCGTAA | *phzB* gene self-complementation |
| C*phzB*-R1 | GGCGGCATCCTCCTTAGTTGG |  |
| C*phzB*-F2 | *ACTAAGGAGGATGCCGCC*ATGCCTAATAGCGCAGCGCA |  |
| C*phzB*-R2 | tggcggccgctctagaTTAAGTTGGGATGCCTTCGCG |  |
| *phzF*-F1 | ccggggatcctctagaGGCTACGACCTGGTCATCAT | *phzF* gene deletion |
| *phzF*-R1 | ACAAGTCATCGGCGTCAAAG |  |
| *phzF*-F2 | *TTGACGCCGATGACTTGT*CGAAGTATCAGGCAATGGCG |  |
| *phzF*-R2 | ggccagtgccaagcttTGGCCTGAACATCCTTGAGC |  |
| *TrpE*-F1 | *CCGGGGATCCTCTAGA*CGACACCCTCAAATGGCTG | *TrpE* gene deletion |
| *TrpE*-R1 | GCAGGAATTCTTCGCGGAT |  |
| *TrpE*-F2 | *TCCGCGAAGAATTCCTGC*GCAGACGGCCGATAGCTGA |  |
| *TrpE*-R2 | ggccagtgccaagcttGGAAAAAGGCGACACCAGC |  |
| *Nat*-F1 | ccggggatcctctagaCGGAGACACCTGGGGGACTT | *Nat* gene deletion |
| *Nat*-R1 | CGCGAAGTCTTCGGCGAAT |  |
| *Nat* -F2 | *TTCGCCGAAGACTTCGCG*CGAAGTTCATCAGGTGGGGCT |  |
| *Nat* -R2 | ggccagtgccaagcttGGAGAAAAACATCCCGCATACC |  |
| *lon*-F1 | ccggggatcctctagaCGAGCTGGATGAGGCTGCGT | *lon* gene deletion |
| *lon*-R1 | GGCAATTCGATGGTTGTCTTCA |  |
| *lon*-F2 | *GACAACCATCGAATTGCC*ACGCATTAGTACGCATTAGCCT |  |
| *lon*-R2 | ggccagtgccaagcttTCTAACCGGCTGCCCTACCG |  |
| *rsmE*-F1 | ccggggatcctctagaAGCACCCGATCCTTGCCGT | *rsmE* gene deletion |
| *rsmE*-R1 | CTTTGCGGGTGAGTATCAGCAT |  |
| *rsmE*-F2 | *GATACTCACCCGCAAAG*CCGGACAAACGCGAAACACC |  |
| *rsmE*-R2 | ggccagtgccaagcttTGCAGGTAGTCGACGTTGGCCT |  |
| *pykA*-F1 | ccggggatcctctagaAAGATCGTTACAACGCGGTCG | *pykA* gene deletion |
| *pykA*-R1 | TGGTACGACGGACGGACATG |  |
| *pykA*-F2 | *TGTCCGTCCGTCGTACCA*ACCCAATGGTCTGAGCCACC |  |
| *pykA*-R2 | ggccagtgccaagcttCGAGTTCGGTTCCAGCCTG |  |
| PET-PhzB-F | GGAATTCCATATGATGCCTAATAGCGCAGCGC | PhzB expression |
| PET-PhzB-R | CCGCTCGAGTTAAGTTGGGATGCCTTCGC |  |
| PET-PhzF-F | GGAATTCCATATGATGCACCATTACGTCATCATCG | PhzF expression |
| PET-PhzF-R | CCGCTCGAGTCATAGAACGATGGTCCCCC |  |
| PET-TrpE-F | GGAATTCCATATGATGATCCGCGAAGAATTCCTGC | TrpE expression |
| PET-TrpE-R | CCGCTCGAGTCAGCTATCGGCCGTCTGCT |  |
| PET-Nat-F | GGAATTCCATATGATGAGTCAGCCCCACCTGATGA | Nat expression |
| PET-Nat-R | CCGCTCGAGTCAGGCCGGTTGCGGCAG |  |

*The lowercase indicates that they overlapped and were used to construct plasmids by the In-fusion method. The italic capital letter indicates the overlapped fragments between Upstream and downstream homologous arms. The red capital letter indicates the enzyme cut site.

**Figure S1** The MS/MS spectrum and the physical form of the purified compound. (A): The MS/MS of standard (up) and the purified compound (down). (B): The physical form of the purified compound


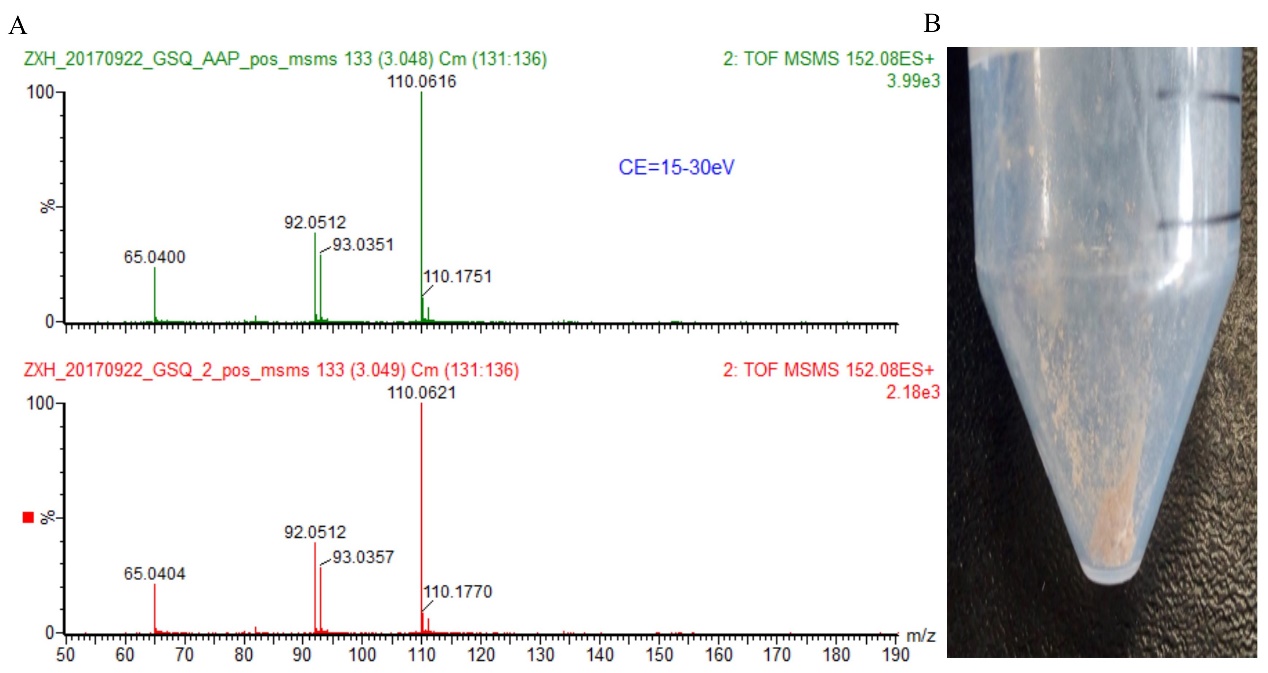


**Figure S2** The ^1^H NMR spectra of 2-acetamidophenol. (DMSO, 600 MHz)


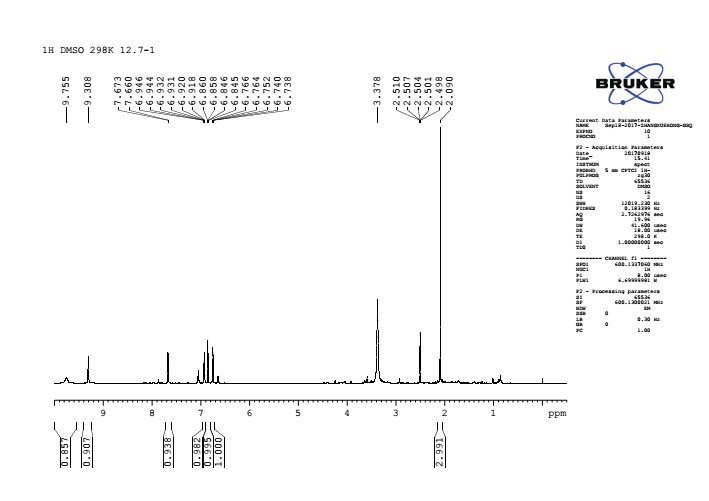


**Figure S3** The ^13^C spectrum of spectra of 2-acetamidophenol (DMSO, 151 MHz)


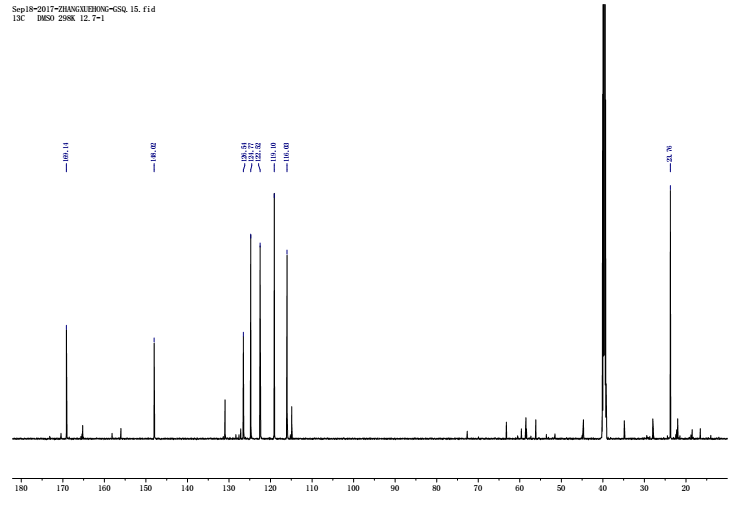


**Figure S4** The HMBC spectrum of 2-acetamidophenol (DMSO, 151 MHz)


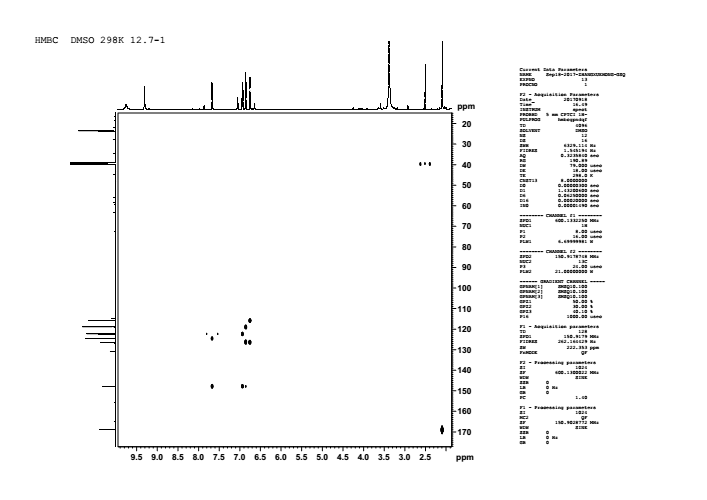


**Figure S5** The HSQC spectrum of 2-acetamidophenol (DMSO, 151 MHz)


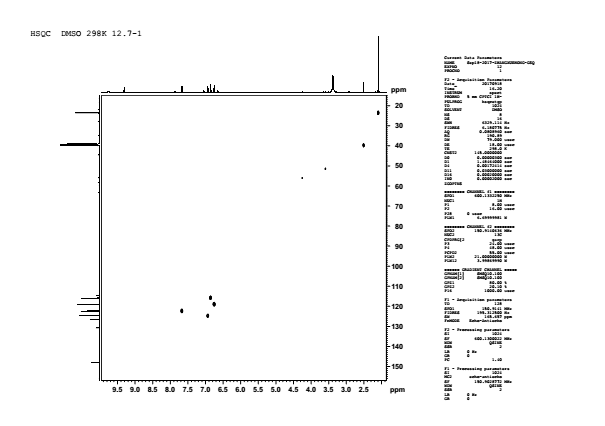


**Figure S6** The NOESY spectrum of 2-acetamidophenol (DMSO, 400 MHz)


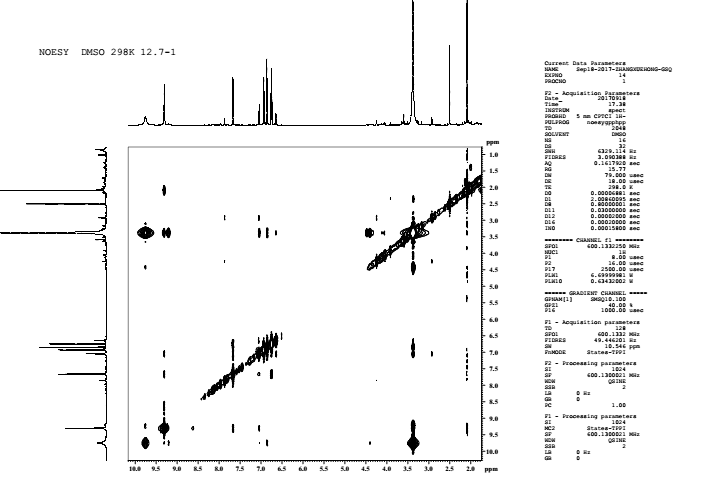


**Figure S7** The COSY spectrum of 2-acetamidophenol (DMSO, 600 MHz)


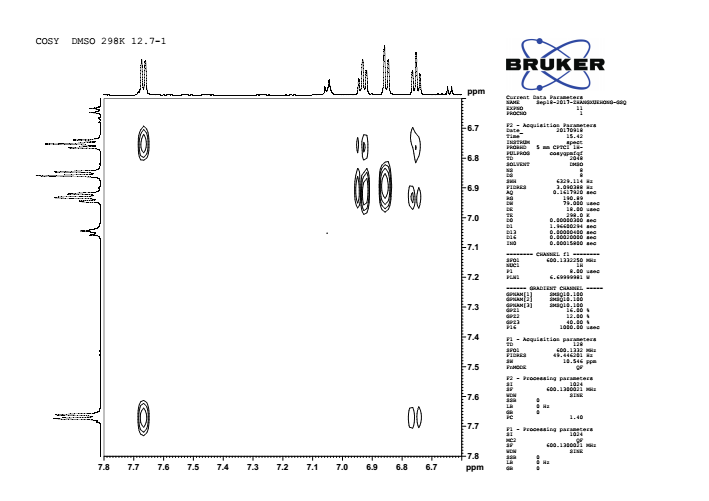


**Figure S8** Linear correlation of AAP and PCA in HT66 derived strains


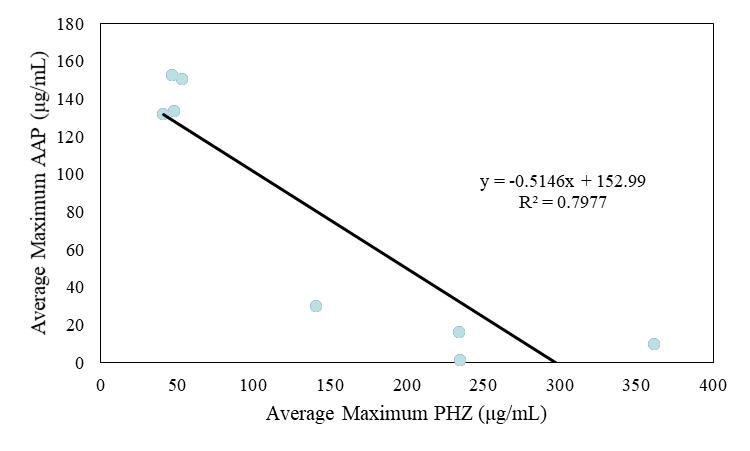


**Figure S9** The protein sequence alignment of arylamine N-acetyltransferase from different strains


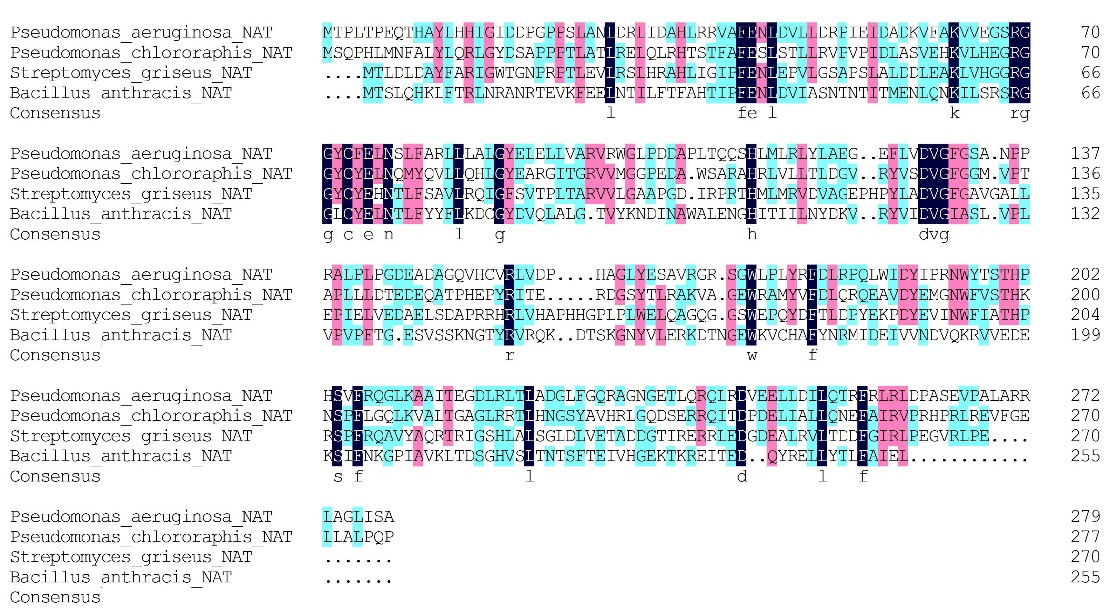


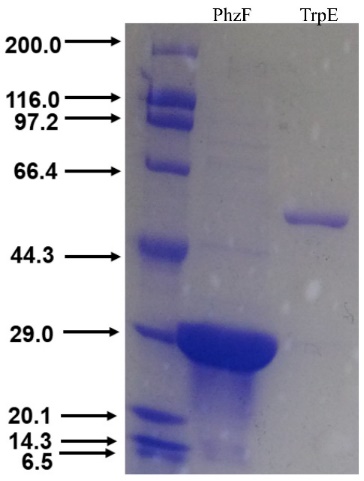
**Figure S10** The protein purification of PhzF and TrpE in *Pseudomonas chlororaphis* HT66

6.1.5 Elucidation of AAP biosynthesis pathway *P. chlororaphis* HT66
